# Survival Benefit of Statin with Anti-Angiogenesis Efficacy in Lung Cancer-Associated Pleural Fluid through FXR Modulation

**DOI:** 10.3390/cancers14112765

**Published:** 2022-06-02

**Authors:** Chen-Liang Tsai, Chih-Ying Changchien, Ying Chen, Chine-Rui Lai, Tzu-Min Chen, Hsin-Han Chang, Wen-Chiuan Tsai, Yu-Ling Tsai, Hao-Chung Tsai, Hung-Yi Lin, Chieh-Yung Wang, Ming-Sheng Shen, Yu-Huei Lin

**Affiliations:** 1 Division of Pulmonary and Critical Care Medicine, Department of Internal Medicine, Tri-Service General Hospital, National Defense Medical Center, Taipei 114, Taiwan; doc10376@gmail.com (C.-L.T.); ssbn621@gmail.com (H.-Y.L.); marechaparis@gmail.com (C.-Y.W.); 2 Department of Internal Medicine, Tri-Service General Hospital, National Defense Medical Center, Taipei 114, Taiwan; koala8072@yahoo.com.tw; 3 Department of Biology and Anatomy, National Defense Medical Center, Taipei 114, Taiwan; ychen0523@mail.ndmctsgh.edu.tw (Y.C.); ray42904917@gmail.com (C.-R.L.); ctm999322@gmail.com (T.-M.C.); albertchang1008@gmail.com (H.-H.C.); 4 Department of Pathology, Tri-Service General Hospital, National Defense Medical Center, Taipei 114, Taiwan; ab95057@hotmail.com (W.-C.T.); c909228@gmail.com (Y.-L.T.); 5 Division of Chest Medicine, Department of Internal Medicine, Tri-Service General Hospital Songshan Branch, Taipei 105, Taiwan; petstsai@yahoo.com.tw; 6 Department of Internal Medicine, Taichung Armed Force General Hospital, Taichung 411, Taiwan; darkevilalien@gmail.com; 7 Post-Baccalaureate Program in Nursing, College of Nursing, Taipei Medical University, Taipei 110, Taiwan

**Keywords:** malignant pleural effusion, non-small cell lung cancer, lung cancer-associated pleural fluid, endothelium, bile acid, FXR, statin

## Abstract

**Simple Summary:**

Our previous works showed that pleural fluid from lung cancer significantly induced endothelial proliferation, migration, and angiogenesis. Since endothelial metabolism was a key step in angiogenesis, we investigated the role of bile acid signaling and FXR expression in pleural angiogenesis. Elevated bile acid levels in lung-cancer-associated pleural fluid (LCPF) were characterized with positive FXR staining in pleural microvessels. We then confirmed the inhibitory effect of an FXR antagonist on LCPF-induced endothelial migration and angiogenesis. Due to the elevated protein expression in the cholesterol metabolism caused by LCPF, lipid-lowering agents with the efficacy needed to counteract LCPF-regulated angiogenesis were evaluated. Statin showed the potent efficacy needed to suppress LCPF-induced endothelial proliferation, migration, and angiogenesis through FXR inhibition. Following that, Kaplan–Meier analysis showed the survival benefit of statin exposure in patients with lung adenocarcinoma with LCPF. Our results suggest that targeting endothelial FXR signaling with statin treatment could ameliorate the angiogenesis activity of LCPF.

**Abstract:**

Lung cancer-related pleural fluid (LCPF) presents as a common complication with limited treatment. Beyond its function in lipid digestion, bile acid was identified as a potent carcinogen to stimulate tumor proliferation. Previous research indicated a correlation between serum bile acid levels and the risk of developing several gastrointestinal cancers. Our study identified elevated bile acid levels in LCPF and increased farnesoid X receptor (FXR) expression as bile acid nuclear receptors in pleural microvessels of lung adenocarcinoma. Additionally, LCPF stimulated the expression of proteins involved in bile acid synthesis and cholesterol metabolism in HUVECs including CYP7A1, StAR, HMGCR, and SREBP2. LCPF-induced endothelial motility and angiogenesis were counteracted by using β-muricholic acid as an FXR antagonist. Moreover, we investigated the efficacy of cholesterol-lowering medications, such as cholestyramine, fenofibrate, and atorvastatin, in regulating LCPF-regulated angiogenesis. Along with suppressing endothelial proliferation and angiogenesis, atorvastatin treatment reversed cholesterol accumulation and endothelial junction disruption caused by LCPF. Statin treatment inhibited LCPF-induced endothelial FXR expression as well as the downstream proteins RXR and SHP. Based on the positive findings of suppressing endothelial angiogenesis, our group further incorporated the effect of statin on clinical patients complicated with LCPF. A Kaplan–Meier analysis revealed the clinical benefit of statin exposure in patients with lung adenocarcinoma with LCPF. Conclusively, our study demonstrated the ability of statin to alleviate LCPF-induced angiogenesis in patients with LCPF via FXR modulation.

## 1. Introduction

Not only do bile acids (BAs) influence cholesterol metabolism via synthesis and excretion, but they have also recently been identified as signaling molecules in the tumor microenvironment [1,2]. The first and rate-limiting enzyme in bile acid production was cholesterol 7-hydroxylase (CYP7A1) [3]. BAs have been implicated in the development of malignancies in the digestive system and extradigestive organs such as the prostate, breasts, and lungs [4]. The advancement of research has increased our understanding of BAs as tumor promoters or suppressors [5,6]. BAs have been shown to operate in cancer cells through modulating nuclear receptors, most notably the farnesoid X receptor, FXR [7]. FXR acted as a bile acid-activated transcription factor, assisting in the maintenance of cholesterol homeostasis [8]. To regulate downstream genes, the retinoid X receptor (RXR) formed a heterodimer with the FXR [9]. Additionally, one of the downstream targets of FXR activation was a small heterodimer partner (SHP) [10]. Numerous medicines targeting FXR activation or inhibition in cardiovascular illness, metabolic disorders, and cancer have been studied [11,12,13]. However, the role of BAs and FXR expression in non-small cell lung cancer (NSCLC) patients with malignant pleural effusions remains unknown.

Our research previously demonstrated enhanced angiogenesis in the pleura milieu of lung adenocarcinoma and the tendency of lung cancer-associated pleural fluid (LCPF) to be angiogenic [14]. Despite recent advances in targeted therapy and chemotherapy, the need for MPE treatment remains unfulfilled [15,16]. Tissue arrays have revealed positive staining on the vasculature of metastatic cancer as well as target tissues of the liver and intestine for FXR expression [17]. The FXR agonist induced endothelial cell motility and angiogenesis in vitro [18]. Additionally, the modulation of endothelial nitric oxide synthase and endothelin-1 by vascular FXR expression has been postulated as a potential target in cardiovascular disease [19]. Thus, the current study sought to determine the efficacy of an FXR modulator in inhibiting the endothelial angiogenesis generated by LCPF.

Apart from its ability to reduce hyperlipidemia, statins have garnered researchers’ interest for their pleotropic effects, particularly in the prevention and treatment of cancer [20,21]. Statins act on transcriptional regulation by interfering with nuclear hormone receptors such as FXR [22]. The protective effect of atorvastatin in mice on a high-fat diet was discovered to be due to the drug’s ability to reset FXR signaling [23]. Additionally, atorvastatin regulates the enterohepatic circulation of BAs by inhibiting FXR mRNA and protein expression [24]. Moreover, clinical analysis showed long-term statin use with better survival in NSCLC patients [21]. However, no retrospective investigations have been conducted to evaluate the effect of statin use in individuals with LCPF. As such, the current in vitro research explored the efficacy of statin in attenuating LCPF-induced angiogenesis as well as the putative control of endothelial FXR signaling.

## 2. Materials and Methods

### 2.1. Kaplan–Meier Analysis of Lung Adenocarcinoma Patients

Clinical data were acquired from the Taipei Medical University Institutional and Clinical Database, which comprises medical records for almost 3 million patients who have visited the Taipei Medical University Hospital, Wan Fang Hospital, and Shuang Ho Hospital. The database contains demographic and clinical information, outpatient and emergency room visits, hospital admissions, laboratory test results, and drug prescriptions; the information has been collected since 1997. The Institutional Review Board ethics committee approved the study (TMU-JIRB No. N202203033).

**Figure 1:**
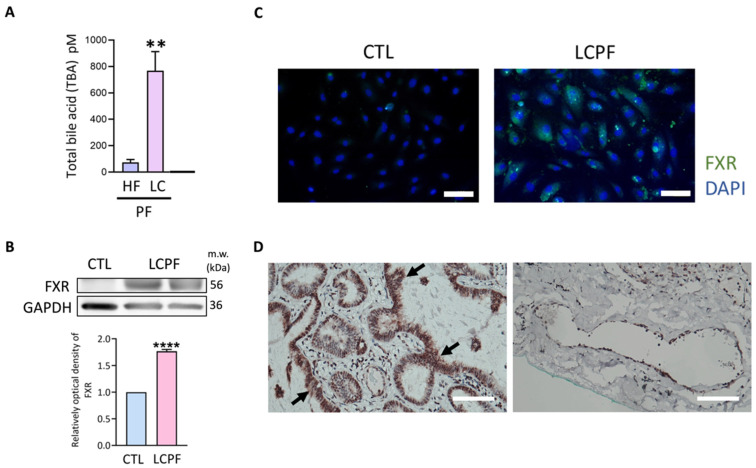
Total bile acid level in pleural fluid and endothelial FXR upregulation by LCPF. (**A**) The total bile acid level was measured by an ELISA kit. Pleural fluid from heart failure patients is referred to as HFPF. The bar graph shows the results of HFPF and LCPF, respectively. Next, HUVECs were incubated with LCPF or a control medium for 24 h. (**B**) FXR protein expression was examined by Western blotting. GAPDH was used as an internal control. (**C**) HUVECs were subjected to immunofluorescence staining for FXR (green) and DAPI (blue). (**D**) Representative images of lung adenocarcinoma (left) and pleural microvessel (right) stained positively for FXR. The magnification is 200× for (**C**) and 100× for (**D**), respectively. Scale bar = 100 μm. The arrow indicates a tumor. ** *p* < 0.01 and **** *p* < 0.0001 compared to the control group.

A retrospective clinical-based cohort study was conducted on stage IV NSCLC patients with and without statin use between January 2010 and December 2019 to validate the outcome. The primary outcome measures were all-cause mortality. To examine the robustness of the primary study’s findings, we conducted sensitivity analyses that only included lung cancer-related mortality. The individuals included met the following criteria, which are illustrated in Figure 1: (1) age: 30 years or older; (2) clinical stage IV and pathologic diagnosis of NSCLC; (3) presentation of malignant pleural effusion; (4) clinical data availability (i.e., sex, age, height, weight, smoking status, drinking status, lipid profiles, comorbidities, and the use of cholesterol-lowering drugs).

The index date is the date of the initial diagnosis of lung cancer. The medication prescriptions were extracted from the pharmaceutical data using the World Health Organization’s Anatomical Therapeutic Chemical (ATC) classification (https://www.whocc.no) (Accessed on 25 March 2022). Statins were defined as medications prescribed within the two years preceding the index date (ATC: C10AA, C10B). The Charlson Comorbidity Index (CCI) score was developed to predict patient mortality and is now the most widely used method in epidemiological studies for adjusting for confounding due to the presence of comorbidities. The CCI was used in this study to adjust for comorbidities defined by the CDMF ICD-9 and ICD-10 scoring systems [25]. To improve the diagnostic validity of the comorbidities included in this study, only those diagnosed at least twice at clinic or hospital visits more than 30 days apart prior to the two-year index date were included. The comorbidity burden was accounted for in this study by categorizing patients into one of three groups based on the CCI (detailed description shown in Table 1) [26].

The checklist for improving the reporting of observational studies in epidemiology (STROBE) was used to guide the reporting of this clinical observational study (Figure S1). Age, sex, gender, smoking status, BMI, and laboratory results, including lipid profiles, pathologic classification, and driver mutation genes, were all potential risk factors for the outcome.

### 2.2. Patient Characteristics and Collection of Pleural Fluid Samples

Pleural fluid samples were acquired using sonography-guided thoracentesis from patients with NSCLC or heart failure who provided written informed consent. Twenty NSCLC patients and five heart failure patients were recruited. From each patient, the drained amount of pleural fluid was often more than 500 mL, and we collected, in total, 5 mL of pleural fluid for use in the in vitro experiments that followed. Fresh samples were immediately centrifuged at 1000× *g* for 15 min and filtered (0.22 µm; Millipore, Burlington, MA, USA) to obtain a cell-free specimen. All samples were stored at −80 °C and thawed once before use.

### 2.3. Hematoxylin and Eosin (HE) Staining and Immunohistochemistry

Pleural tissues were fixed in 10% *v*/*v* formalin, embedded in paraffin, and sectioned at 6 μm on a microtome. The paraffin sections were deparaffinized and stained with HE in a standard manner to assess general tissue morphology. For immunohistochemical staining, pleural tissues were fixed in phosphate-buffered saline (PBS; 137 mM NaCl, 2.7 mM KCl, 1.5 mM KH_2_PO_4_, and 8 mM Na_2_HPO_4_ pH 7.4) with 10% *v*/*v* formaldehyde, 4% *w*/*v* sucrose, and 0.15 mM CaCl_2_, incubated with permeabilization buffer (PBS with 0.2% *v*/*v* Triton X-100, or PBST) and blocked with blocking buffer (PBST with 5% *w*/*v* nonfat milk). After antigen retrieval, pleural tissues were incubated with primary antibody against rabbit FXR (1:200 dilution, Millipore) and secondary goat anti-rabbit antibody (Jackson ImmunoResearch Laboratories, Inc., West Grove, PA, USA).

### 2.4. Culture of Primary Endothelial Cells

HUVECs were purchased from the Bioresource Collection and Research Center (BCRC, Taiwan) and cultured in endothelial cell medium (ScienCell Research Laboratories, Carlsbad, CA, USA).

### 2.5. Drugs and Reagents

Dimethyl sulfoxide (DMSO), 3-(4,5-dimethylthiazol-2-yl)-2,5-diphenyltetrazolium bromide (MTT), and Coomassie brilliant blue G-250 were purchased from Sigma-Aldrich (St. Louis, MO, USA). β-Muricholic acid (MCA), GW4064, atorvastatin, fenofibrate, and cholestyramine were purchased from Sigma.

### 2.6. Cell Survival Assay

HUVECs were plated at a density of 2 × 10^4^ per well in a 96-well plate. Next, HUVECs were cultured with 30% LCPFF (*v*/*v*) or control medium. After 24 h incubation, the cells were washed with PBS, 0.5 mg/mL MTT was added, and the plates were incubated for another 4 h. Cells were then lysed with DMSO. The absorbance was measured at 590 nm for each well.

### 2.7. Transwell Assays

HUVECs were seeded in the upper chamber of a Transwell^®^ plate (Corning Costar, Cornyn, NY, USA) at a density of 2 × 10^4^ per well. After incubation with LCPF for 16 h, those that migrated to the lower chamber were fixed with 10% *v*/*v* formalin, washed with PBS, and stained with Coomassie brilliant blue G-250. Migrated cells in five randomly selected fields from each membrane were examined from six independent experiments.

### 2.8. Tube Formation Assay

Matrigel (50 mL/well) was added to a prechilled 96-well plate and incubated for 1 h at 37 °C. Then, HUVECs (1 × 10^4^) were seeded into each well with either 30% MAPF (*v*/*v*) or control medium. After 12 h of incubation, tube formation was imaged. Then, the cellular networks of angiogenesis were quantified and analyzed with ImageJ including tube length, branch point, and tube width.

### 2.9. Western Blotting

HUVECs were rinsed once with PBS and lysed with 60 mM PIPES (piperazine-N,N′-bis (2-ethanesulfonic acid)), 25 mM HEPES *N*-(2-hydroxyethyl)piperazine-N′-(2-ethanesulfonic acid), 0.15% Triton X-100, 10 mM ethylene glycol-bis(β-aminoethyl ether)-N,N,N′,N′-tetraacetic acid, 2 mM magnesium chloride, 1 mM sodium fluoride, 2.5 mM sodium pyrophosphate, 1 mM phenylmethylsulfonyl fluoride, 1 mM sodium orthovanadate, 1 mM β-glycerophosphate, 1 μg/mL leupeptin, 1 μg/mL pepstatin A, and 1 μg/mL aprotinin (pH 6.9). Forty micrograms of each sample was separated by 10% sodium dodecyl sulfate-polyacrylamide gel electrophoresis (SDS-PAGE); then, the proteins were transferred to a nitrocellulose membrane (Bio-Rad) according to the manufacturer’s instructions. The membranes were incubated overnight at 4 °C with primary antibodies in Tris-buffered saline with Tween (TBST) (50 mM Tris-HCl, 150 mM sodium chloride, and 0.1% *v*/*v* Tween-20, pH 7.4). The primary antibodies were specific for glyceraldehyde 3-phosphate dehydrogenase (GAPDH, Cell Signaling Technology, Danvers, MA, USA), Cyp7A1(Santa Cruz, Dallas, TX, USA), FXR (Santa Cruz, USA), HMGCR (Santa Cruz, USA), PPARγ (Cell Signaling Technology, USA), RXR (Cell Signaling Technology, USA), SHP (Santa Cruz, USA), SREBP1 (Santa Cruz, USA), SREBP2 (Santa Cruz, USA), and StAR (Santa Cruz, USA). After the membranes were washed, the strips were incubated with a 1:5000 or 1:10,000 dilution of horseradish peroxidase-conjugated anti-rabbit IgG from Cell Signaling Technology. Next, the blots were treated with a chemiluminescent substrate developing solution (Bio-Rad). Band densities were captured and quantified by densitometry using ImageJ. GAPDH served as a loading control for immunoblotting analysis. The control sample was set as 100%, and LCPF-cultured samples were normalized to the control.

### 2.10. Immunofluorescence Staining

HUVECs were seeded on coverslips and incubated in the presence of LCPF for 8 h. Cells were then rinsed with PBS and fixed with 10% *v*/*v* formalin in PBS (pH 7.4). A blocking solution (5% milk in 0.1% *v*/*v* Triton X-100) was applied to prevent nonspecific binding. Primary antibody against VE-cadherin (Cell Signaling Technology, USA) in blocking buffer was incubated with the HUVECs at 4 °C overnight. After the antibody was washed, the slides were incubated with fluorescein isothiocyanate-conjugated goat anti-mouse and anti-rabbit IgG (Sigma-Aldrich) for 1 h. Finally, coverslips were mounted with a mounting medium (Gel Mount Aqueous, Sigma) and photographed with a Nikon D1X digital camera (Carl Zeiss, Oberkochen, Germany). For filipin cholesterol staining, HUVECs were fixed with 4% (*w*/*v*) paraformaldehyde in PBS for 30 min and stained with 50 μg/mL filipin in PBS at room temperature for 2 h. Cells were then washed with PBS three times and mounted.

### 2.11. Statistical Analysis

Data are expressed as the averages of at least six samples and are presented as the mean ± standard error of the mean (SEM). Analysis was performed using a Student’s *t*-test, with a *p*-value of <0.05 chosen to indicate statistical significance. In the databased section, the chi-squared test and Student’s t-test were utilized to compare subject characteristics. The Kaplan–Meier method was used to estimate the survival probability for the two groups, which were compared with the log-rank test. The Cox proportional hazards regression model was used to assess the impact of multiple risk factors on all-cause mortality and lung cancer-related mortality of NSCLC patients. All analyses were performed using SAS 9.3 software (SAS Institute Inc., Cary, NC, USA). All tests were two-tailed; the alpha level of significance was set to *p* < 0.05.

## 3. Results

### 3.1. Bile Acid Abundance in LCPF and Upregulation of Bile Acid Nuclear Receptor FXR Signaling in Endothelial Cells by LCPF

In pleural fluid analyses, LCPF had a 10-fold rise in bile acid levels compared to heart failure samples (Figure 1A). FXR was identified as a bile acid nuclear receptor, and LCPF-cultured HUVECs increased FXR expression (Figure 1B). LCPF caused positive FXR staining in endothelial cells’ nuclei and cytoplasm (Figure 1C). The HUVEC results revealed the ability of LCPF to upregulate endothelial FXR expression. Correspondingly, immunohistochemistry sections of lung adenocarcinoma had positive FXR staining in both tumor cells and endothelial cells (Figure 1D). The above results revealed the abundance of bile acid in LCPF and endothelial FXR expression induced by LCPF.

**Figure 2:**
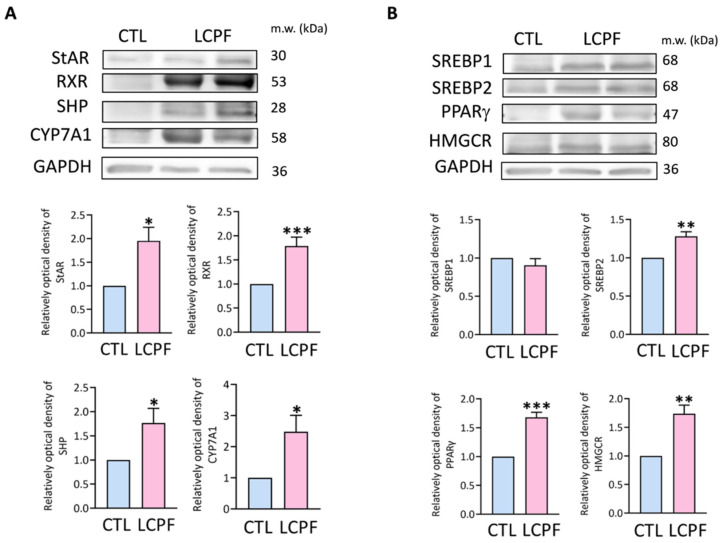
Upregulation of bile acid synthesis and cholesterol metabolism protein by LCPF stimulation. HUVECs were incubated with LCPF or control medium for 24 h. (**A**) Proteins regulating bile acid synthesis, such as steroidogenic acute regulatory protein (StAR), RXR, SHP, and CYP7A1, were examined by Western blotting. GAPDH was used as an internal control. (**B**) Proteins responsible for cholesterol metabolism were analyzed including sterol regulatory element binding protein 1 (SREBP1), SREBP2, peroxisome proliferator-activated receptor gamma (PPAR-γ), and HMG-CoA reductase (HMGCR). GAPDH was used as an internal control. * *p* < 0.05; ** *p* < 0.01; *** *p* < 0.001, compared to the control group. Original blots are shown in Figure S3.

The steroidogenic acute regulatory protein (StAR) and CYP7A1 enzymes were important in the synthesis of bile acids. RXR and SHP worked along with FXR to modulate bile acid signaling. Following FXR activation, LCPF increased protein expression of StAR, CYP7A1, RXR, and SHP in HUVECs, implying that FXR signaling was active (Figure 2A). FXR signaling and cholesterol metabolism were linked in a complicated way. We also looked at the protein levels of sterol regulatory element binding protein 1 (SREBP1), SREBP2, peroxisome proliferator-activated receptor gamma (PPAR-γ), and HMG-CoA reductase (HMGCR). The SREBP2, PPAR-γ, and HMGCR levels were found to be elevated in HUVECs grown with LCPF (Figure 2B). Our findings not only identified substantial bile acid in LCPF, but also demonstrated LCPF’s ability to stimulate FXR signaling and cholesterol metabolism in HUVECs.

### 3.2. Efficacy of FXR Antagonist and Cholesterol-Lowering Drugs in Attenuating LCPF-Induced Endothelial Angiogenesis

**Figure 3:**
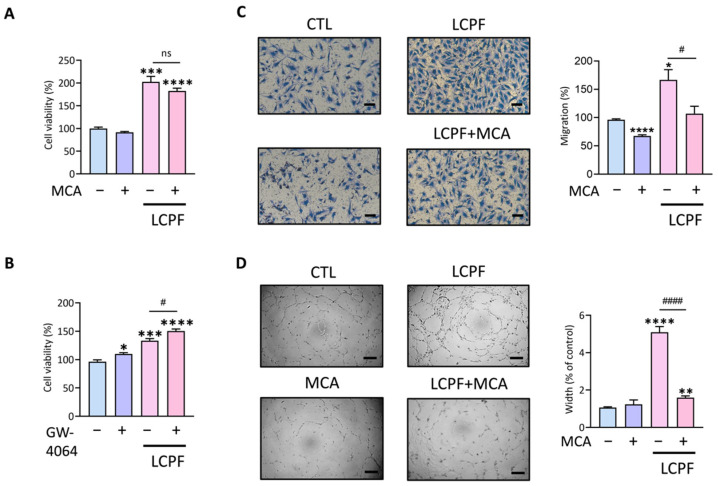
β-Muricholic acid as an FXR antagonist alleviated LCPF-induced endothelial motility and angiogenesis. β-Muricholic acid (MCA) and GW4064 were applied as the FXR antagonist and agonist, respectively. HUVECs were treated with MCA (10 µM) or GW4064 (10 µM) in a LCPF-containing medium for 24 h. (**A**,**B**) Cell viability was analyzed by MTT assay after treatment. (**C**) Representative images and statistical analysis of a Transwell assay at 18 h after LCPF culture with or without 10 µM MCA. (**D**) Micrographs and statistical analysis of tube width at 12 h after LCPF culture with or without 10 µM MCA. Scale bar = 200 μm. * *p* < 0.05; ** *p* < 0.01; *** *p* < 0.001; **** *p* < 0.0001, compared to the control group. # *p* < 0.05; #### *p* < 0.0001, compared to the LCPF group.

We examined β-muricholic acid (MCA) as an FXR antagonist in LCPF-regulated angiogenesis because of LCPF-induced FXR overexpression in HUVECs. GW-4064 was used as an FXR agonist. MCA treatment had little effect on the viability of LCPF-upregulated HUVECs (Figure 3A). GW4064, on the other hand, boosted endothelial cell viability in the LCPF-treated group (Figure 3B). Cotreatment with E- or Z-guggulsterone had a synergistic effect on HUVEC viability when combined with LCPF (Figure S1). The findings above demonstrate the importance of FXR activation in LCPF-regulated angiogenesis. The experiments that followed looked at the efficacy of MCA as an FXR antagonist on endothelial motility and angiogenesis. MCA treatment inhibited LCPF-induced HUVEC migration and angiogenesis in Transwell and tube formation assays (Figure 3C,D). We established the justification for FXR blockage to suppress LCPF-induced angiogenesis based on the findings above.

**Figure 4:**
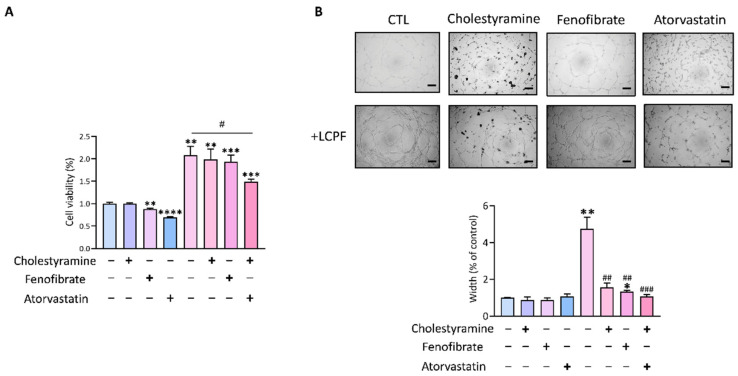
Effect of lipid-lowering medications on LCPF-induced endothelial viability and angiogenesis. HUVECs were treated with cholestyramine (20 µM), fenofibrate (25 µM), or atorvastatin (20 µM) in a LCPF-containing medium. (**A**) Cell viability was analyzed by MTT assay after 24 h of treatment. (**B**) Representative images and analysis of tube formation after HUVECs cultured in the above conditions for 12 h are shown. Scale bar = 200 μm. * *p* < 0.05; ** *p* < 0.01; *** *p* < 0.001; **** *p* < 0.0001, compared to the control group. # *p* < 0.05; ## *p* <0.01; ### *p* < 0.001, compared to the LCPF group.

The link between FXR signaling and cholesterol metabolism had previously been proposed [8,27]. Following that, we looked at the effects of cholesterol-lowering drugs, such cholestyramine, fenofibrate, and atorvastatin, on LCPF-regulated angiogenesis [28]. Only statin treatment significantly reduced LCPF-upregulated endothelial proliferation in the MTT experiment (Figure 4A). Three cholesterol-lowering medications inhibited LCPF-induced HUVEC angiogenesis in a tube formation assay (Figure 4B). Our findings showed that FXR antagonists and statins inhibited LCPF-induced angiogenesis.

### 3.3. Statin Effects to Compensate LCPF-Induced Endothelial Cholesterol Accumulation, Cell Junction Disruption

**Figure 5:**
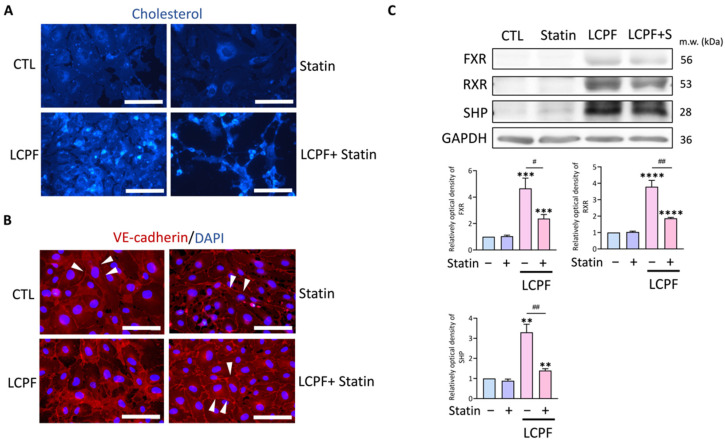
Effect of statin on cellular cholesterol, endothelium junction, and FXR signaling. HUVECs were treated with or without 20 µM atorvastatin (statin) in the presence of LAPF for 24 h. (**A**,**B**) HUVECs were processed for immunofluorescence staining of cholesterol (blue) and VE-cadherin (red), respectively. Nuclei are visualized with DAPI. The arrowheads indicate the distribution of VE-cadherin. The magnification is 400×. Scale bar = 100 μm. (**C**) Proteins in FXR signaling, including FXR, RXR, and SHP, were examined by Western blotting. GAPDH was used as an internal control. ** *p* < 0.01; *** *p* < 0.001; **** *p* < 0.0001, compared to the control group. # *p* < 0.05 and ## *p* < 0.01, compared to the LCPF group. Original blots are shown in Figure S3.

The involvement of cholesterol metabolism and junction integrity in endothelial angiogenesis manipulation seemed critical. According to immunofluorescence labeling, LCPF enhanced the cholesterol content in HUVECs considerably (Figure 5A). Statins alleviated the aforementioned phenomenon. The basic junctional protein VE-cadherin was responsible for the maintenance of the endothelial cell–cell barrier. There was a loss of VE-cadherin localization in the endothelium periphery after 24 h of LCPF therapy (Figure 5B). Statin treatment shifted the distribution of VE-cadherin back to cell–cell interaction. The retinoid X receptor (RXR) and its small heterodimer partner (SHP) worked in tandem with the FXR to modulate bile acid signaling. In HUVECs, LCPF increased the protein expression of FXR, RXR, and SHP, but statin cotreatment reduced the aforementioned trends (Figure 5C). Furthermore, statin inhibited the expression of LCPF-induced integrins β1 and β3 and VEGFR1 and -2 in endothelial cells (Figure S2). Our findings show that statins control LCPF-induced angiogenesis and junction disruption via FXR inhibition.

### 3.4. Clinical Effect of Statin Use on NSCLC Patients with Malignant Pleural Effusion

**Figure 6:**
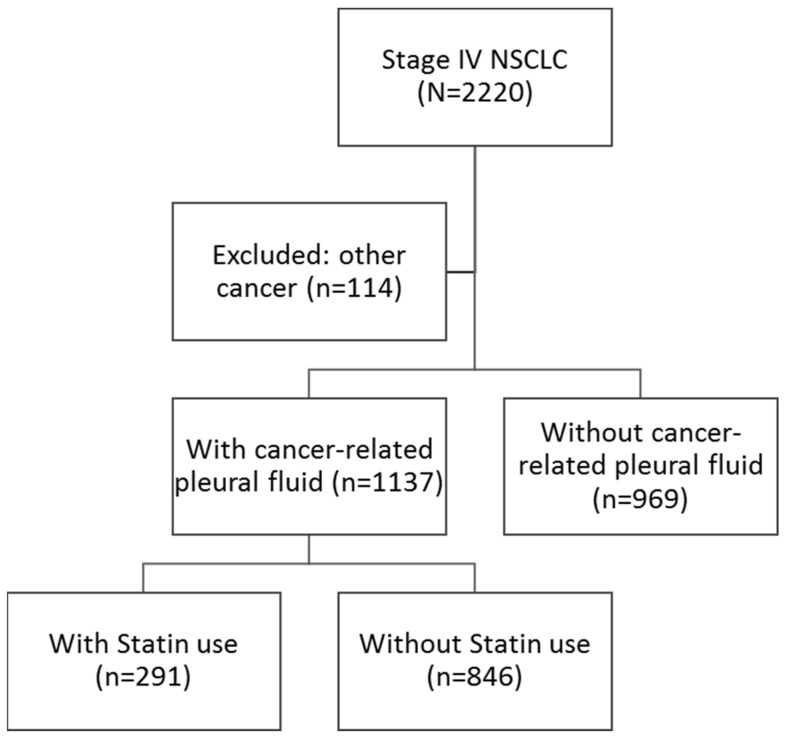
Study flow of NSCLC stage IV patients with cancer-related pleural effusion.

**Figure 7:**
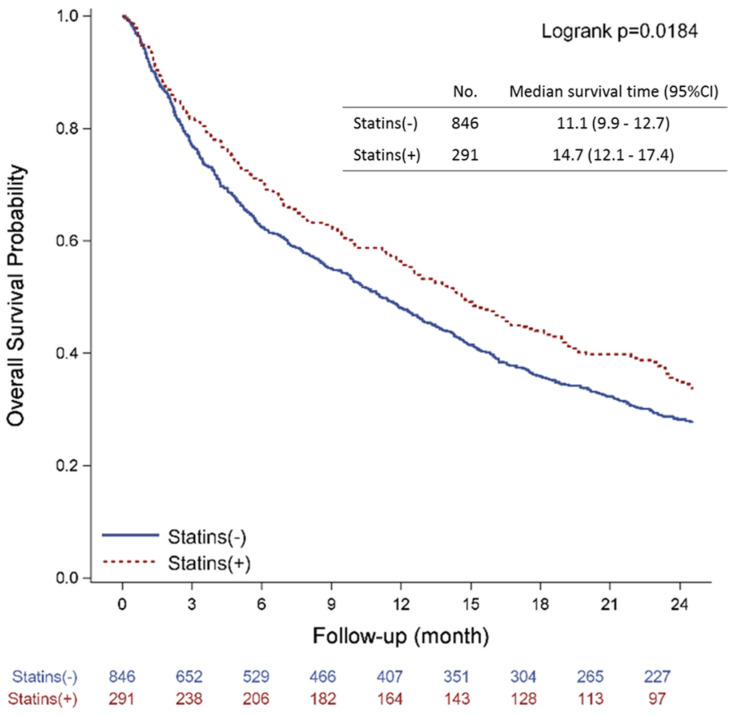
Kaplan–Meier plot of all-cause mortality among NSCLC stage IV patients with cancer-related pleural effusion, with and without statin use, over 24 months.

In addition to statin’s anti-angiogenesis effect in vitro, we investigated statin’s potential benefit in NSCLC cancer patients. After excluding patients with other tumors, those without cancer-related pleural effusion and those with incomplete demographic and clinical data, 1137 NSCLC patients with LCPF were identified (Figure 6). The demographics of those who enrolled are detailed in Table 1. A Kaplan–Meier plot of all-cause mortality in LCPF patients exposed with and without stain over a 24 month period is shown in Figure 7. Patients who received statins had a median overall survival (OS) of 14.7 months, compared to 11.1 months for those who did not receive statins. We then used the Cox proportional-hazards model to calculate all-cause and lung cancer-related mortality in Table 2. The crude model had no adjustments, whereas the adjusted model was adjusted for age, gender, BMI, CCI, TC, LDL-C, and EGFR to minimize confounder effects. The control group consisted of NSCLC stage IV patients who had cancer-related pleural effusion but did not take statins (risk of 1). The adjusted all-cause mortality hazard ratio for statin users was 0.69 when compared to the control group (95% CI, 0.51–0.92). The adjusted lung cancer mortality rate in patients taking statins was 0.63. (95% CI, 0.46–0.86). We also provided statement checklist of STROBE (Table S1). Patients who did not take statins had a higher risk of not only all-cause mortality but also lung cancer-related mortality than those who did.

## 4. Discussion

Beyond its role as a digestive surfactant, bile acid has been implicated in cancer cell proliferation, metastasis, and tumor angiogenesis [2]. Elevated serum bile acid levels have been identified as a significant risk factor for gastrointestinal cancers [4,25]. However, the role of circulating bile acid in NSCLC, particularly in pleural fluid, had not been reported. Our findings established the presence of bile acid in LCPF, indicating that it may be a druggable target in the pleural microenvironment. Compared with HFPF, the bile acid level of LCPF was significantly higher. However, the composition between exudative and transudative pleural fluid was different including the content of albumin, lactate dehydrogenase (LDH), and cholesterol [29]. The above variables might contribute to the difference in bile acid levels between HFPF and LCPF. Future study of bile acid should also quantify other exudative pleural effusion such as inflammatory etiology. FXR expression was significantly increased in NSCLC as a nuclear bile acid receptor, and FXR knockdown inhibited lung cancer growth both in vitro and in vivo [30]. A previous study revealed FXR as a functional protein in the vasculature of metastatic cancers [17]; moreover, FXR modulated endothelial cell motility via FAK and MMP9 suppression [18]. In pancreatic cancer, FXR downregulation resulted in decreased VEGFA mRNA transcription through impaired DNA-binding activity of NF-κB [31]. Our results demonstrate that FXR may play a role in pleural angiogenesis with the regulation of FAK and VEGFA. Additionally, LCPF incubation was capable of inducing bile acid synthesis and cholesterol metabolism protein expression in HUVEC. RXR, StAR, SREBP2, and HMGCR expression in endothelial cells contributed to dysregulated lipid metabolism and exacerbated atherosclerosis progression [32,33,34]. Our study demonstrated the emergence of bile acid and cholesterol homeostasis during lung cancer pleural angiogenesis.

Numerous malignancies, including lung cancer, have been studied using FXR blockade [35]. In addition, MCA was identified as a naturally occurring FXR antagonist produced by the gut microbiota [36]. The purpose of this study was to examine the efficacy of MCA as an FXR antagonist in alleviating LCPF-regulated endothelial motility and angiogenesis. These findings not only established the role of FXR in pleural angiogenesis but also supported the future use of FXR antagonists in the treatment of MPE. Moreover, cholesterol metabolic reprogramming has been extensively studied in the context of cancer promotion [37]. Lipid-lowering medications were therefore repurposed for cancer treatment [38]. Atorvastatin, fenofibrate, and cholestyramine all inhibited LCPF-induced angiogenesis in a tube formation assay, implying the possibility of combining lipid-lowering drugs to treat MPE. Additional clinical trials of antihyperlipidemic agents are required to determine the outcome of NSCLC patients with MPE.

Endothelial cholesterol metabolism has previously been linked to aberrant angiogenesis [39]. In HUVECs, LCPF increased cholesterol loading, and statin treatment abolished LCPF-induced angiogenesis. Previous research regarded the cholesterol loading in vascular endothelium as part of the pathogenesis during atherosclerosis [40]. However, cholesterol supplementation could promote VEGFR2-induced angiogenesis through regulating lipid rafts formation where VEGFR2 are anchored [41]. Serum samples of coronary artery disease patients showed decreased VEGF levels in an atorvastatin-treated group [42]. The above results further provide a possible mechanism of LCPF-induced angiogenesis through cholesterol and FXR signaling. Along with multiple growth factors, bile acid abundance may contribute to LCPF-mediated angiogenesis and serve as a therapeutic target. In colon carcinogenesis, cholesterol metabolism and bile acid signaling were closely coordinated [43,44]. As an inhibitor of HMG-CoA reductase, statin also influenced the bile acid pool via FXR modulation [23,45]. In the aspect of angiogenesis, statin was found to induce endothelial apoptosis and angiostatic effect through geranylated proteins. These observations could interpret the local effect of statin in pleural angiogenesis. Our findings suggest that statin has the potential to inhibit LCPF-regulated angiogenesis by suppressing FXR and downstream signaling. The role of statins in the prevention and treatment of NSCLC has been extensively discussed but not in the survival of MPE patients [46]. From observational studies of NSCLC, statin treatment was correlated with decreased risk of mortality and the improvement of patient survival but not overall response rate [47]. More specifically, a nationwide population-based study revealed statins had the potential to amplify the treatment response of lung cancer patients receiving EGFR-TKI therapy [21]. For NSCL patients treated with nivolumab, statin use could increase the response rate and prolong time-to-treatment failure [48]. In addition to the positive results of statin in terms of suppressing LCPF, our analysis of MPE patients treated with and without stain revealed a survival benefit. Along with the beneficial effects of statin on LCPF suppression, our analysis of MPE patients exposed to stain treatment revealed a survival benefit. The current study established the therapeutic use of statins in MPE patients through bench and clinical data.

## 5. Conclusions

Our study is the first to establish the presence of bile acid in pleural fluid and the upregulation of the bile acid receptor FXR in pleural microvessels. Furthermore, LCPF treatment increased bile acid synthesis and cholesterol metabolism protein expression in endothelial cells including CYP7A1, StAR, HMGCR, and SREBP2. FXR inhibition was shown to be effective at suppressing LCPF-regulated endothelial angiogenesis. Along with ameliorating the angiogenesis induced by LCPF, statin treatment reversed cholesterol accumulation and the endothelial barrier disruption caused by LCPF. Additionally, the analysis of NSCLC patients indicated that statin use was beneficial in the MPE subgroup. However, the effects of statins on survival benefit have some limitations. The underlying dyslipidemia is the primary cause for the patient’s statin exposure. Even after adjusting the LDL level in the subsequent analysis, we still discovered a survival advantage in the group that was exposed to stains. This was the result of a retrospective cohort observation. To confirm the survival benefit of statins in advanced NSCLC with pleural effusion, more clinical trials are required. The current study established the role of bile acid signaling in pleural angiogenesis and demonstrated the usefulness of statin as an FXR antagonist in suppressing LCPF in vitro and in vivo.

## Figures and Tables

**Table 1:** Characteristic of NSCLC stage IV patients with PE (*n* = 1137).

Variable	NSCLC Stage IV with PE, *n* (%)		*p*-Value
	Statin (+) (*n* = 291)	Statin (−) (*n* = 846)	
**TNM stage**			
**T**			0.99
T0-2	64 (22.0)	188 (22.2)	
T3	38 (13.1)	112 (13.2)	
T4	189 (64.9)	546 (64.5)	
**N**			0.79
N0-1	72 (24.7)	193 (22.8)	
N2	73 (25.1)	220 (26.0)	
N3	146 (50.2)	433 (51.2)	
**M**			0.10
M1a	174 (59.8)	459 (54.3)	
M1b	117 (40.2)	387 (45.7)	
**Age, years (mean ± SD)**	70.9 ± 12.6	67.3 ± 14.0	<0.0001
**Gender**			0.20
Male	151 (51.9)	476 (56.3)	
Female	140 (48.1)	370 (43.7)	
**BMI, kg/m^2^**			0.0012
≤25	201 (69.1)	664 (78.5)	
>25	90 (30.9)	182 (21.5)	
**Smoking status**			0.87
No	187 (64.3)	539 (63.7)	
Ever/current	104 (35.7)	307 (36.3)	
**Alcohol consumption**			0.79
No	236 (81.1)	680 (80.4)	
Yes	55 (18.9)	166 (19.6)	
**CCI**			<0.0001
0	126 (43.3)	603 (71.3)	
1–2	67 (23.0)	137 (16.2)	
3+	98 (33.7)	106 (12.5)	
**Lipid profiles, mg/dL**			
**Total cholesterol, mg/Dl (mean ± SD)**	200.1 ± 53.1	179.2 ± 44.2	<0.0001
Normal or low (<200)	107 (36.8)	141 (16.7)	
High (≥200)	84 (28.9)	50 (5.9)	
**LDL-C, mg/dL (mean ± SD)**	125.3 ± 37.8	109.5 ± 31.2	0.0002
**HDL-C, mg/dL (mean ± SD)**	51.0 ± 14.2	49.8 ± 16.6	0.57
**Pathological type**			0.41
Squamous carcinoma	34 (11.7)	108 (12.8)	
Adenocarcinoma	243 (83.5)	711 (84.0)	
Others	14 (4.8)	27 (3.2)	
**Driver genes**			
EGFR	144 (49.5)	334 (39.5)	0.0029
ALK	5 (1.7)	12 (1.4)	0.72

PE, pleural effusion; ALK, anaplastic lymphoma kinase; BMI, body mass index; CCI, Charlson Comorbidity Index; EGFR, epidermal growth factor receptor; HDL-C, high-density lipoprotein cholesterol; LDL-C, low-density lipoprotein cholesterol; NSCLC, non-small cell lung cancer; TC, total cholesterol; TG, triglyceride; TNM, tumor-node-metastasis; SD, standard deviation.

**Table 2:** Cox proportional-hazards model of all-cause mortality and lung cancer-related mortality among NSCLC stage IV patients with cancer-related pleural effusion with and without statin use.

No.		Events (%)		Crude HR (95% CI)			*p*-Value	Adjusted HR (95% CI)			*p*-Value
**All-cause mortality**											
Statin (−)	846	619	(73.2%)	1.0	(Ref.)			1.0	(Ref.)		
Statin (+)	291	194	(66.7%)	0.84	(0.73–0.97)		0.0172	0.69	(0.51–0.92)		0.0131
**Lung cancer-related mortality**											
Statin (−)	846	593	(70.1%)	1.0	(Ref.)			1.0	(Ref.)		
Statin (+)	291	187	(64.3%)	0.86	(0.74–0.99)		0.0373	0.63	(0.46–0.86)		0.0038

Adjusted for age, gender, BMI, CCI, TC, LDL-C, and EGFR. BMI, body mass index; CCI, Charlson Comorbidity Index; CI, confidence interval; EGFR, epidermal growth factor receptor; HR, hazard ratio; LDL-C, low-density lipoprotein cholesterol; NSCLC, non-small cell lung cancer; TC, total cholesterol.

## Data Availability

The data presented in this study are available on request from the corresponding author.

## Supplementary Materials

The following supporting information can be downloaded at: https://www.mdpi.com/article/10.3390/cancers14112765/s1, Figure S1: Effect of E and Z isomer of guggulsterone (GS) on LCPF-regulated endothelial proliferation; Figure S2: Changes in integrin β1 and -3 and in VEGFR1 and -2 protein levels when HUVECs were cotreated with LCPF and statin. Figure S3: Original bolts. Table S1: STROBE statement checklist.
