# Supplementary material for: Survival Benefit of Statin with Anti-Angiogenesis Efficacy in Lung Cancer-Associated Pleural Fluid through FXR Modulation
Source: Cancers (Basel). 2022 Jun 2;14(11):2765. doi: 10.3390/cancers14112765 (PMC9179389; doi:10.3390/cancers14112765)
Supplement: Supplementary file 1 [file cancers-14-02765-s001.zip › cancers-1714842-supplementary.pdf]

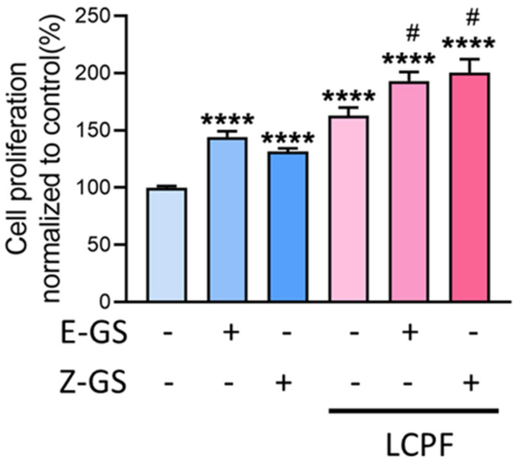

Supplementary Figure S1. Effect of E and Z isomer of guggulsterone (GS) on LCPF-regulated endothelial proliferation. (**A**) HUVEC cells were treated with E- or Z-GS, in LCPF-containing medium for 24 h. Both concentrations of E- and Z-GS were 10 μM. Cell viability was analyzed by MTT assay. ****p < 0.0001 compared to the control group. #p < 0.05, compared to the LCPF group.

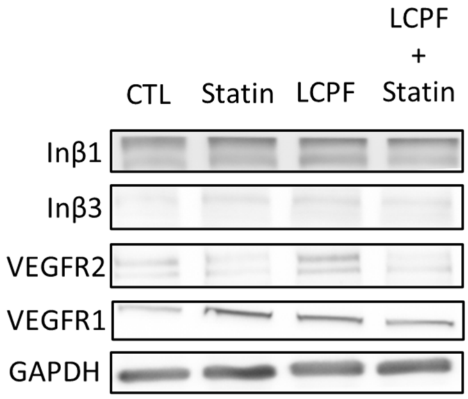

Supplementary Figure S2. Changes in integrin β1 and 3, and VEGFR1 and 2 protein levels when HUVECs were cotreated with LCPF and statin. HUVECs were treated with or without 20 μM atorvastatin (statin), in the presence of LAPF, for 24 h.

(**A**) Protein expression of integrin β1 and 3, and VEGFR1 and 2 was evaluated by Western blotting. GAPDH was used as the internal control.

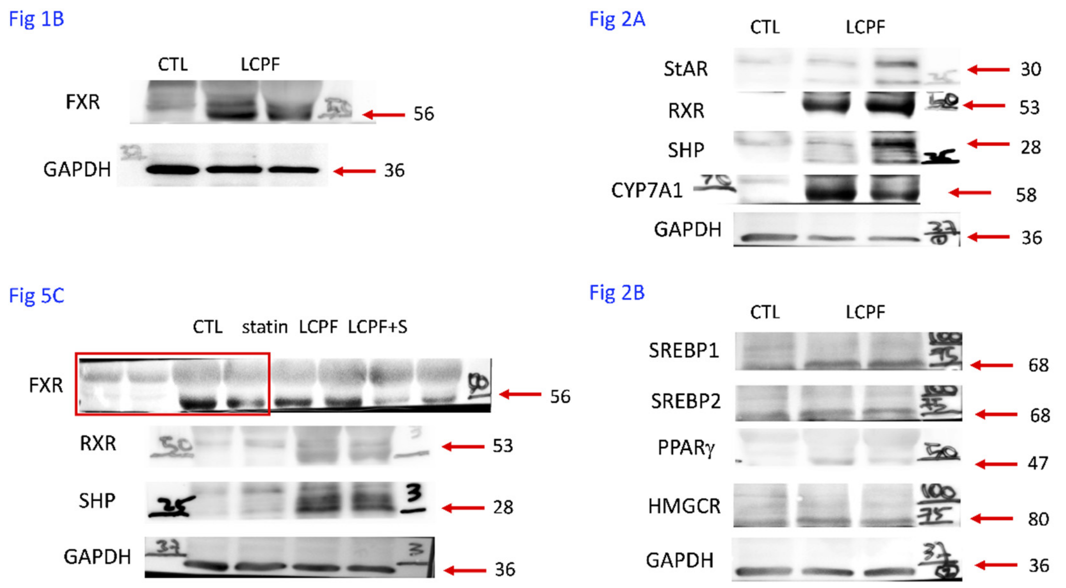

Supplementary Figure S3. Original blots of Fig. 1B, 2A, 2B and 5C.

Supplementary Table S1. STROBE statement checklist

		Reporting Item	Page Number
**Title and abstract**			
Title	#1a	Indicate the study's design with a commonly used term in the title or the abstract	P1
Abstract	#1b	Provide in the abstract an informative and balanced summary of what was done and what was found	P1-2
**Introduction**			

Background / rationale	#2	Explain the scientific background and rationale for the investigation being reported	P3
Objectives	#3	State specific objectives, including any prespecified hypotheses	P3
**Methods**			
Study design	#4	Present key elements of study design early in the paper	P3-6
Setting	#5	Describe the setting, locations, and relevant dates, including periods of recruitment, exposure, follow-up, and data collection	P3, P5
Eligibility criteria	#6a	Give the eligibility criteria, and the sources and methods of case ascertainment and control selection. Give the rationale for the choice of cases and controls. For matched studies, give matching criteria and the number of controls per case	P5, P10, Figure 6
Eligibility criteria	#6b	For matched studies, give matching criteria and the number of controls per case	P10, Figure 6
	#7	Clearly define all outcomes, exposures, predictors, potential confounders, and effect modifiers. Give diagnostic criteria, if applicable	P10
Data sources / measurement	#8	For each variable of interest give sources of data and details of methods of assessment (measurement). Describe comparability of	P5-6

		assessment methods if there is more than one group. Give information separately for cases and controls.	
Bias	#9	Describe any efforts to address potential sources of bias	P10
Study size	#10	Explain how the study size was arrived at	P10
Quantitative variables	#11	Explain how quantitative variables were handled in the analyses. If applicable, describe which groupings were chosen, and why	P5-6
Statistical methods	#12a	Describe all statistical methods, including those used to control for confounding	P6, P10
Statistical methods	#12b	Describe any methods used to examine subgroups and interactions	No
Statistical methods	#12c	Explain how missing data were addressed	P10
Statistical methods	#12d	If applicable, explain how matching of cases and controls was addressed	NA
Statistical methods	#12e	Describe any sensitivity analyses	P10
**Results**			
Participants	#13a	Report numbers of individuals at each stage of study—eg numbers potentially eligible, examined for eligibility, confirmed eligible, included in the study, completing follow-up,	P10, Figure 6

		and analysed. Give information separately for cases and controls.	
Participants	#13b	Give reasons for non-participation at each stage	Figure 6
Participants	#13c	Consider use of a flow diagram	Figure 6
Descriptive data	#14a	Give characteristics of study participants (eg demographic, clinical, social) and information on exposures and potential confounders. Give information separately for cases and controls	Table1
Descriptive data	#14b	Indicate number of participants with missing data for each variable of interest	Figure 6
Outcome data	#15	Report numbers in each exposure category, or summary measures of exposure. Give information separately for cases and controls	P10, Figure 6
Main results	#16a	Give unadjusted estimates and, if applicable, confounder-adjusted estimates and their precision (eg, 95% confidence interval). Make clear which confounders were adjusted for and why they were included	P10, Table 2
Main results	#16b	Report category boundaries when continuous variables were categorized	Table1
Main results	#16c	If relevant, consider translating estimates of relative risk into absolute risk for a meaningful time period	Figure 7

Other analyses	#17	Report other analyses done—e.g., analyses of subgroups and interactions, and sensitivity analyses	P10
**Discussion**			
Key results	#18	Summarise key results with reference to study objectives	P14-15
Limitations	#19	Discuss limitations of the study, taking into account sources of potential bias or imprecision. Discuss both direction and magnitude of any potential bias.	P14-15
Interpretation	#20	Give a cautious overall interpretation considering objectives, limitations, multiplicity of analyses, results from similar studies, and other relevant evidence.	P13-14
Generalisability	#21	Discuss the generalisability (external validity) of the study results	P13-14
**Other Information**			
Funding	#22	Give the source of funding and the role of the funders for the present study and, if applicable, for the original study on which the present article is based	P15

Abbreviations: STROBE, The strengthening the reporting of observation studies in epidemiology.
